# Online interventions for problem gamblers with and without co-occurring mental health symptoms: Protocol for a randomized controlled trial

**DOI:** 10.1186/s12889-016-3291-7

**Published:** 2016-07-22

**Authors:** John A. Cunningham, David C. Hodgins, Kylie Bennett, Anthony Bennett, Marina Talevski, Corey S. Mackenzie, Christian S. Hendershot

**Affiliations:** Centre for Addiction and Mental Health, 33 Russell St., Toronto, ON M5S 2S1 Canada; Australian National University, Canberra, Australia; University of Toronto, Toronto, Canada; University of Calgary, Calgary, Canada; University of Manitoba, Winnipeg, Canada

**Keywords:** Clinical trial, Randomized controlled trial, Brief intervention, Gambling disorders, Comorbidity, Depression, Anxiety, Online intervention, Internet intervention, Trial protocol

## Abstract

**Background:**

Comorbidity between problem gambling and depression or anxiety is common. Further, the treatment needs of people with co-occurring gambling and mental health symptoms may be different from those of problem gamblers who do not have a co-occurring mental health concern. The current randomized controlled trial (RCT) will evaluate whether there is a benefit to providing access to mental health Internet interventions (G + MH intervention) in addition to an Internet intervention for problem gambling (G-only intervention) in participants with gambling problems who do or do not have co-occurring mental health symptoms.

**Methods:**

Potential participants will be screened using an online survey to identify participants meeting criteria for problem gambling. As part of the baseline screening process, measures of current depression and anxiety will be assessed. Eligible participants agreeing (*N* = 280) to take part in the study will be randomized to one of two versions of an online intervention for gamblers – an intervention that just targets gambling issues (G-only) versus a website that contains interventions for depression and anxiety in addition to an intervention for gamblers (G + MH). It is predicted that problem gamblers who do not have co-occurring mental health symptoms will display no significant difference between intervention conditions at a six-month follow-up. However, for those with co-occurring mental health symptoms, it is predicted that participants receiving access to the G + MH website will display significantly reduced gambling outcomes at six-month follow-up as compared to those provided with G-only website.

**Discussion:**

The trial will produce information on the best means of providing online help to gamblers with and without co-occurring mental health symptoms.

**Trial registration:**

ClinicalTrials.gov NCT02800096; Registration date: June 14, 2016.

## Background

It is estimated that up to half of pathological gamblers have co-occurring mental health symptoms (e.g., depression or anxiety disorder) [1–6]. While there is little work in this area, co-occurring mental health symptoms are thought to impact on the treatment needs of problem gamblers [7–12]. The interrelationship between problem gambling and co-occurring mental health symptoms may not have the same cause for all those experiencing these co-occurring conditions. For some, gambling may be an attempt to alleviate the symptoms of depression and anxiety [13]. Others may experience symptoms of anxiety and depression as a result of increasing difficulties with gambling (financial or otherwise) [14]. For others, while occurring simultaneously, problem gambling and co-occurring mental health symptoms may not be causally related (or both might be the result of some third factor) [15]. In addition, it is important to consider the extent to which the interrelationship between problematic gambling and co-occurring mental health symptoms varies by gender – both in the strength of the observed relationship as well as in the underlying mechanisms that are driving this interrelationship [2, 16]. Further research employing longitudinal designs is needed to develop a better understanding of these interrelationships [5, 12, 17].

While the functional relationship of gambling to co-occurring mental health symptoms is an important research question, the goal of the present trial is a pragmatic one and, as such, does not need to wait upon a better understanding of these interrelationships. We seek to determine whether providing simultaneous access to online help for gambling problems and mental health symptoms is of benefit for those experiencing both concerns (and, to a lesser extent, that providing simultaneous access is not disadvantageous for those with just a gambling problem and no co-occurring depression or anxiety symptoms). A secondary goal will be to determine if there are moderators (e.g., gender, extent of use) for the hypothesized benefit of providing the G + MH intervention website to those problem gamblers with co-occurring mental health symptoms.

### The need for alternatives to face-to-face care

The large majority of problem gamblers will never access traditional treatment [18–20]. Barriers include stigma, availability, and a desire for self-reliance [21]. The Internet is widely available and has been recognized as an important platform through which to decrease treatment-seeking stigma, and to provide evidence-based care in an accessible and cost-efficient fashion [22]. Despite being unwilling, or unable to attend traditional treatment, many problem gamblers have voiced an interest in accessing help through other means, such as the Internet [23].

### Self-help for gambling

There is a growing evidence base for self-help interventions targeting gambling – primarily through bibliotherapy with or without limited contact with a therapist [24, 25]. To increase the accessibility of such interventions, some efforts have also been made to provide Internet-based self-help materials for problem gamblers [26, 27]. However, these online interventions have little or no published evidence base and relevant issues, such as the best way to provide such services to people with co-occurring mental health symptoms have, as yet, not been addressed.

## Major research questions

The proposed trial will compare two Internet intervention websites – an intervention that just targets gambling issues (G-only) versus one that contains interventions for anxiety and depression in addition to an intervention for gamblers (G + MH). The primary hypotheses are:

- **Hypothesis 1:** For problem gamblers with co-occurring mental health symptoms, it is predicted that participants provided access to the G + MH website will display significantly reduced gambling outcomes at three- and six-month follow-ups as compared to those provided access to the G-only website.
- **Hypothesis 2:** Problem gamblers without co-occurring mental health symptoms will display no significant difference between the G-only and G + MH websites at three- and six-month follow-ups.
- **Hypothesis 3:** Respondents who have more involvement with the G-only intervention between baseline and three-month follow-up will demonstrate more improvement in gambling outcomes at six-month follow-up, compared to respondents who have less involvement with the G-only intervention.

## Methods/design

### Study design

**Fig. 1:**
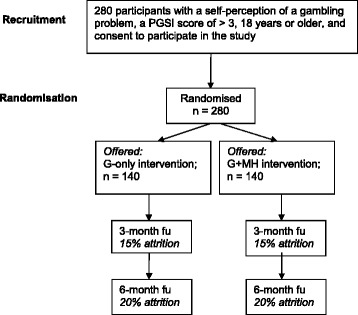
Overview of the proposed intervention trial

The proposed study is a two-arm, double blinded, parallel group RCT. See Fig. 1 below for a Consort Diagram summarizing this trial design.

### Recruitment

Potential respondents will be recruited using online advertisements (e.g., Google adwords). The advertisement will ask if the person is concerned about their gambling and if they are interested in participating in a study that contains online help for gambling (with compensation provided). Interested participants will follow a link to a brief online survey to assess eligibility (details provided below). Those eligible and who provide informed consent will be sent an email with a unique link to the remaining baseline assessment. Participants who complete the baseline measures will then be randomized into one of the two Internet interventions described below (G-only versus G + MH). Only participants who complete the baseline survey via their unique link will be randomized and included in the trial. After completing the baseline survey, participants create a password which they then use to access the respective intervention websites through the study website portal. Research staff involved in the trial will not be informed of respondents’ group allocation during interventions or at follow-up. All participants will be followed-up at three- and six-month post-randomization using an online survey (an email invitation including a unique link will be sent to each participant). In order to promote retention, participants completing each of the follow-ups will be sent a $20 gift certificate from Amazon.ca (i.e., honorarium of $40 total). Ethics approval was obtained from the Research Ethics Boards of the Centre for Addiction and Mental Health (Canada) and the Australian National University (Australia).

#### Inclusion criteria

Primary inclusion criteria will follow those used in the ongoing trial by Hodgins et al. [26] and comprise being 18 years of age or older, perception of a gambling problem and scoring 3 or greater on the Problem Gambling Severity Index (PGSI) [28]. Prior treatment access will be measured but will not be used as an inclusion/exclusion criterion. This is because the intent of this trial is to evaluate the impact of the interventions in the extended range of potential community participants. Random assignment to condition with stratification will ensure that socio-demographic characteristics, such as treatment access, will be evenly distributed across conditions. Similarly, hazardous alcohol use and illicit drug consumption will be measured but will not be used as exclusion criteria. Finally, the trial will recruit participants with and without co-occurring mental health symptoms. We anticipate, based on prevalence data, that approximately 50 % of the sample will be experiencing co-occurring mental health symptoms. This will be established using the Kessler 10, with a score of 22 more indicating current psychological distress [29, 30].

### Randomization

Randomization will be conducted via an automated computer algorithm, set up by a researcher not involved in the day-to-day conduct of the trial according to ICH Guideline E9 [31]. Randomization will be stratified by age (18–35 years/over 36 years), sex (male/female), and prior use of treatment for problem gambling (have previously accessed treatment/have not accessed treatment).

### Interventions

#### G-only

The gambling only Internet intervention will consist of a new online version of the self-change tools developed by Hodgins et al. [32]. These tools have shown a significant impact on gambling in three trials of the paper-based version of these materials [33–35], and have previously been translated successfully into an online format [26]. A major focus is to provide individuals with clear and concise behavioural and cognitive strategies for meeting the goal of reducing or quitting gambling. The various workbook sections are readily adaptable into online interactive formats.

#### G + MH

For participants in the G + MH condition, logging into the web portal will allow them to access the G-only Internet intervention as well as an online intervention for depression and anxiety. The mental health intervention chosen is MoodGYM, an extensively evaluated intervention found to be effective in a variety of different settings [36–38].

### Baseline assessment

The online assessment includes a demographic profile (age, gender, education, marital status, income, employment status) and a gambling, mental health and treatment history assessment. Problem gambling severity will be measured using the past year PGSI and the past three month version of the NORC DSM-IV Screen for Gambling Problems (NODS) which indicates DSM-IV severity [39, 40]. Hodgins [41] administered the NODS to problem gamblers as part of a 1-year follow-up after a brief treatment to assess its utility as a treatment outcome measure. Internal reliability was fair to good and the factor structure and item-total correlations supported the existence of a single higher order construct that correlated moderately with gambling behaviour and outcome. Prior treatment access will be measured using the items developed for previous trials conducted by Hodgins et al. [33, 34]. In addition, participants will be asked to identify a treatment goal (quit or reduced gambling) and how successful they think they will be (0 “not at all” to 10 “extremely”) in the next three months and in the next six months.

Severity of depressive symptoms will be measured using the PHQ-9 [42]. Severity of anxiety symptoms will be measured using the GAD-7 [43]. The Kessler 10 (K10) questionnaire will be included to provide a continuous measure of general psychological distress that is responsive to change over time. The K10 has been well validated and its brevity and simple response format are attractive features. It also produces a summary measure indicating probability of currently experiencing an anxiety or depressive disorder [29, 30].

Hazardous alcohol consumption will be measured using the Alcohol Use Disorder Identification Test Consumption measure (AUDIT-C) [44]. Illicit drug use will be assessed in a manor commonly used on general population surveys – by asking if the participant had used (from a list) any of the primary illicit drug categories ever, and in the last 12 months [45]. A more detailed assessment of illicit drug use is not warranted in this situation as the incidence of use will be too low for detailed analysis.

Quality of life will be assessed by the WHOQoL-8, an eight-item version of a widely used measure. This short form has been used in a number of countries, is robust psychometrically, and overall performance is strongly correlated with scores from the original WHOQoL [46].

### Follow-up assessments

Three and six months after randomization, an email invitation will be sent to participants containing a link to the follow-up assessment. Up to two reminder emails will be sent to promote retention in the trial. The follow-up will consist of an assessment of gambling behaviour, problem gambling severity (NODS), self-rated improvement, psychiatric distress, alcohol and illicit drug use, quality of life, and use of other treatment resources. Primary outcome measures will consist of problem gambling severity (as measured by the NORC DSM-IV screen for Gambling Problems (NODS – past 3-month version) [41], and mean days per month gambling in the past 3 months.

#### Use of interventions

We will have access to a complete record of the amount and type of use participants make of the G-only and G + GM interventions. This information will be used to test the moderation hypothesis that degree of involvement with the online gambling intervention is related to success at overcoming gambling problems. We will operationalize degree of involvement with G-only and G + MH interventions by recording the number of times the participant accesses the site as well as the number of modules completed and length of involvement with the site (e.g. use of the site over time).

### Power analysis

We propose to collect a sample of 280 participants and we estimate that we will successfully follow about 224 participants at six months (20 % attrition) [34]. This number will also provide sufficient power to conduct the proposed statistical tests comparing hypotheses, based upon gambling frequency and NODS data from Hodgins et al. [33, 34], assuming a correlation of .5 between baseline and follow-up values, power = .80 and an α = .05. This sample size will be sufficient to detect a difference of about two gambling days per month between conditions at each follow-up interval. Smaller differences may not be clinically important. Similarly, this sample size will be sufficiently powered to detect a 1 point difference on the NODS at six months. These calculations are based upon a repeated measures ANOVA model. The proposed analyses will employ mixed effects repeated measures models and, as such, will have greater statistical power because all observed data are included.

Also important is to ensure that there are enough females in the sample to conduct the proposed sex difference analyses. Approximately a third of problem gamblers in the general population are female [47]. With the assumption that this proportion would be maintained in our sample (i.e., those who would be concerned about their gambling and agree to participate in the study), roughly 92 of the 280 respondents will be female. A sample with 23 respondents per condition is sufficiently large to conduct the proposed analyses. However, our experience using these same recruitment methods in other studies is that females tend to be more interested in self-help materials than males and also, to be willing to participate in the proposed research. Thus, we fully anticipate that more than a third of respondents will be female (perhaps as many as half will be female if the recruitment trends in our other studies hold true with this trial as well).

### Data analysis

Analyses for Hypotheses 1 and 2, comparing outcomes between groups, will employ mixed effects repeated measures models that use all available data for each participant. Separate analyses will be conducted for each primary outcome variable. This same analytic approach, with the addition of interaction terms, will be used for secondary analyses examining moderators (e.g., extent of use, gender). Missing data will be handled using a maximum likelihood approach to estimate covariances, variances and means.

## Discussion

The large majority of problem gamblers will not access formal treatment [18–20]. Many problem gamblers also suffer from co-occurring depression or anxiety [1–6]. Given the widespread use of the Internet, the provision of online services for problem gamblers has been recognized as one promising means of overcoming many of the barriers to accessing formal treatment as Internet interventions can be accessed in the person’s home or other convenient locations. The current trial will address one question regarding the delivery of Internet interventions. Mainly, for problem gamblers with or without co-occurring mental health symptoms, is there benefit to providing combined access to online interventions for gambling and for depression or anxiety? It is hoped that the results of this trial will shed light on this question as well as advance the research base on Internet intervention for addictions and mental health in general.

## Abbreviations

AUDIT-C, Alcohol Use Disorders Identification Test - Consumption measure; G + MH, G-only intervention plus access to MoodGYM, an Internet intervention for depression and anxiety; GAD-7, Generalized Anxiety Disorder 7 item scale; G-only, internet intervention targeting problem gambling; K10, Kessler Psychological Distress Scale; NODS, The National Opinion Research Centre DSM screen for gambling problems; PGSI, problem gambling severity index; PHQ-9, patient health questionnaire; RCT, randomized controlled trial
